# Impact of biological sex, concussion history and sport on baseline NeuroTracker performance in university varsity athletes

**DOI:** 10.3389/fspor.2024.1372350

**Published:** 2024-11-22

**Authors:** Jean-Michel Acquin, Yannick Desjardins, Alexandre Deschamps, Étienne Fallu, Philippe Fait, Laurie-Ann Corbin-Berrigan

**Affiliations:** ^1^ Department of Human Kinetics, Université du Québec à Trois-Rivières, Trois-Rivières, QC, Canada; ^2^ Groupe de Recherche sur les Affectations Neuromusculosquelettiques (GRAN), Université du Québec à Trois-Rivières, Trois-Rivières, QC, Canada; ^3^ Research Center in Neuropsychology and Cognition (CERNEC), Université de Montréal, Montréal, QC, Canada

**Keywords:** mild traumatic brain injury, perceptual-cognitive skills, preseason baselines, 3D-MOT, multiple object tracking

## Abstract

This study aimed to assess the impact of biological sex, concussion history, and type of sport on the baseline NeuroTracker performance, a test/train three-dimensional multiple object tracking paradigm used in sport contexts, in university level varsity athletes. A total of 136 university level varsity athletes participating in male ice hockey, male or female soccer, female volleyball, and mixed biological sex cheerleading underwent preseason NeuroTracker baseline assessments. Significant differences in NeuroTracker performance were observed based on biological sex (*p* < 0.01) and type of sport played (*p* < 0.05). Male athletes and hockey players demonstrated higher NeuroTracker performance compared to their counterparts. However, no significant differences were found in NeuroTracker performance based on the history of concussion. Thus, factors such as biological sex and type of sport played may influence baseline NeuroTracker performance.

## Introduction

NeuroTracker, a three-dimensional multiple object tracking (3D-MOT) system, operates as a perceptual-cognitive task, engaging working memory, complex motion integration, and distributed attention processing to track multiple objects in time and space (1). Originally designed to enhance perceptual-cognitive skills and sport performance, NeuroTracker has gained popularity over the last decade, serving as both a testing and training methodology in various populations. In individuals with a history of concussion, it has been suggested that NeuroTracker performance may be influenced by state of recovery, where performance in individuals with acute concussion may be diminished and back to normal upon full recovery (2), and could thus be a potential objective tool for clinicians in diagnosis and management. Additionally, NeuroTracker has shown to be a reliable preseason baseline measure of cognitive performance in athletes (3), with attempts to link it to sport-specific abilities (4) and talent identification (5).

However, most studies on the NeuroTracker are performed on small samples of participants, factors that may influence NeuroTracker performance in athletes need to be further studied in order to better access the applicability of the NeuroTracker to discern between different variables (e.g., concussion diagnosis, talent identification, etc.). For instance, the vast majority of NeuroTracker concussion studies have been conducted on pediatric populations (6), whereas talent identification has mostly been studied on male elite athletes (4, 5). It is however now recognized that baseline NeuroTracker performance may be influenced by athletic level and gender (6, 7), where elite athletes and males may exhibit higher NeuroTracker performance, than their female and non-athlete counterparts. Hence, this study aimed to investigate whether individual factors like biological sex, concussion history, and sport type affect baseline NeuroTracker performance in a homogeneous group of university varsity athletes.

## Methods

### Participants

A convenience sample comprising 136 university varsity athletes (44.9% female) participated in the study, with an age range of 18–29 years (mean age: 22.63 ± 2.25 years). Inclusion criteria required participants to be 18 years or older, fluent in French or English, and engaging in one of the following university varsity sports: male ice hockey, male or female soccer, mixed sex cheerleading, and female volleyball. Sports selected for this study were based on both availability of athletes at the time of testing with regards to sporting season (preseason), and accessibility of athletes for recruitment. Exclusion criteria applied to individuals who had experienced a concussion within the last three months, had a been diagnosed with an attention deficit disorder (with or without hyperactivity) or learning disability, had vision problems not corrected by lenses or glasses, or were unable to perceive 3D. The study spanned the school years 2019–2022, and for participants involved in varsity sports for multiple years within this timeframe, data used in this study were derived from the most recent year of data collection. Ethical approval was obtained from the University of Quebec at Trois-Rivières' research ethics committee (CER-14-205-07.17), and informed consent was obtained from all participants.

### Procedure

Prior to the beginning of the regular sporting season, participants underwent a series of baseline assessments, previously detailed by our research group (3), which included the NeuroTracker (CogniSens Inc., Montreal, Canada), as part of a concussion baseline battery. As part of this assessment series, participants completed a demographics questionnaire that gathered information on biological sex, concussion history, and sport participation. Concussion history was reported dichotomously, with participants indicating whether they had previously experienced a concussion. Those with a positive history of concussion then specified the number of concussions they had sustained. The number of previous concussions was subsequently categorized into three groups: 0, 1, and 2 or more concussions (8).

Participants wore active 3D glasses while seated in a dimly lit room, positioned 1.5 meters away from a 60-inch projector displaying the 3D-MOT task. Following standard NeuroTracker baseline performance evaluation (1), the Core (4) mode was employed, consisting of three blocks, each comprising 20 trials, to establish an average baseline NeuroTracker performance. This allowed participants to get accustomed to the task, while limiting learning effects of the task. Completing each block, involving 20 trials, took approximately 6 to 8 min, and mean NeuroTracker performance (average of 3 blocks) was determined within approximately 20 min. The trial sequence encompassed five distinct steps: (1) 8 yellow spheres resembling tennis balls are presented, (2) 4 of the 8 spheres are highlighted and identified as targets for the trial, (3) targets return to their original color and the 8 spheres start moving randomly in the virtual space for 8 s, (4) the 8 spheres stop moving, and the targets must be identified (either verbally to the research assistant, or manually by the participant on a keyboard) and finally (5) feedback on the identification is provided (1).

Every participant started the task at a predetermined standard speed (1.00 m/s), as per Core (4) mode. Then, the speed at which spheres moved in the virtual space during each block was participant-dependent and followed a staircase pattern. Successful trials resulted in a higher speed in the subsequent trial, while unsuccessful trials led to a slower speed in the following trial. Performance was measured in speed thresholds (one speed threshold per block), reflecting the ability to visually track 4 targets at a set speed. A brief two-minute break was provided between blocks.

### Statistical analysis

Descriptive statistics were conducted for participants' demographic information, concussion history, and sport-related details. NeuroTracker performance analysis was defined as mean speed threshold (average of 3 blocks), in line with standard NeuroTracker performance measure as per the Core (4) mode. To compare NeuroTracker performance across biological sex, history of concussion and sport, one-way ANOVAs were performed with significance set at *p* < 0.05. *Post hoc* Bonferroni analyses were performed to identify significant differences in cases where more than two groups were compared. Statistical analyses were performed using IBM SPSS version 28.

## Results

Demographic information for participants is detailed in Table 1. Out of 136 athletes (44.9% female), 66 (48.5%) reported a history of concussion (27 females; 39 males), with 34 athletes having sustained one prior concussion and 32 athletes having sustained two or more concussions. Athletes participated in various sports, including male ice hockey (*n* = 37), male soccer (*n* = 31), female soccer (*n* = 32), female volleyball (*n* = 9), and mixed biological sex cheerleading (*n* = 27). Average NeuroTracker performance across the group was 1.31 ± 0.38 m/s.

**Table 1:** Participants' characteristics.

Sports	Age (mean, SD)	Female athletes (*n* = 60)	Male athletes (*n* = 76)	Concussion history (*n* = 66)
Cheerleading	23.1 ± 2.2	20	7	14
Ice Hockey	23.5 ± 2.1	–	37	22
Soccer	22.2 ± 2.3	31	32	28
Volleyball	22.4 ± 3.4	9	–	2

One-way ANOVAs revealed significant differences for average NeuroTracker performance based on biological sex [*F*(1, 135) = 15.291, *p* < 0.01], with males exhibiting higher speed threshold calculations than females, non-significant differences between history of concussion [*F*(1, 135) = 0.319, *p* = 0.573] and number of previously sustained concussions [*F*(2, 133) = 0.270, *p* = 0.764], and significant differences for type of sport [*F*(4, 132) = 3.312, *p* = 0.013]. Post-hoc Bonferonni analysis suggested that male ice hockey players exhibited higher average NeuroTracker performance than mixed biological sex cheerleaders, but results did not reach significance (*p* = 0.076) (Table 2).

**Table 2:** NeuroTracker performance, reflected in speed thresholds, based on biological sex, concussion history and sport.

	*N* (%)	ST block 1	ST block 2	ST block 3	χ¯ST
Based on biological sex					
Males	77 (56.6)	1.32 ± 0.48	1.45 ± 0.46	1.48 ± 0.49	1.41 ± 0.40
Females	59 (43.4)	1.05 ± 0.38	1.23 ± 0.35	1.21 ± 0.41	1.17 ± 0.30
Based on history of concussion					
History of concussion	66 (48.5)	1.14 ± 0.43	1.32 ± 0.45	1.40 ± 0.47	1.29 ± 0.38
No history of concussion	70 (51.5)	1.26 ± 0.46	1.37 ± 0.41	1.34 ± 0.47	1.32 ± 0.38
Based on number of reported concussions					
0	70 (51.5)	1.26 ± 0.46	1.38 ± 0.41	1.34 ± 0.47	1.33 ± 0.38
1	34 (25.0)	1.11 ± 0.47	1.31 ± 0.40	1.39 ± 0.35	1.28 ± 0.36
2+	32 (23.5)	1.17 ± 0.40	1.31 ± 0.49	1.38 ± 0.58	1.29 ± 0.40
Based on sport					
Ice Hockey (M)	37 (27.2)	1.32 ± 0.42	1.48 ± 0.40	1.51 ± 0.42	1.44 ± 0.33
Soccer (M)	31 (22.8)	1.33 ± 0.55	1.40 ± 0.52	1.48 ± 0.53	1.40 ± 0.48
Soccer (F)	32 (23.5)	1.13 ± 0.36	1.24 ± 0.39	1.25 ± 0.40	1.21 ± 0.31
Cheerleading (M&F)	27 (19.9)	1.06 ± 0.45	1.29 ± 0.38	1.17 ± 0.48	1.19 ± 0.38
Volleyball (F)	9 (6.6)	0.92 ± 0.27	1.17 ± 0.33	1.39 ± 0.43	1.16 ± 0.28
Average (*n* = 136)					1.31 ± 0.38

F, stands for female; M, stands for male; ST, stands for speed threshold.

## Discussion

Our study showed biological sex differences at baseline NeuroTracker evaluation reflected in average performance. Males exhibited higher speed threshold calculations in average performance. These findings align with Legault and colleagues (7), which reported that males had higher scores than females, irrespective of athletic status, in a sample slightly younger than ours. This difference could be explained by inherent neurological and physiological disparities between biological sexes (9), such as males demonstrating higher temporal resolution of attention than females in the general population (10). Given that the majority of NeuroTracker studies involve diverse population pools, we advocate for future studies to diligently control for biological sex.

Contrary to expectations, concussion history did not impact NeuroTracker baseline performance, as no significant differences emerged across the history of concussion and the number of prior concussions reported. While previous research suggested that recent concussions could alter performance in repeated NeuroTracker training (11), individuals clinically recovered from concussions exhibited similar NeuroTracker performance to healthy controls (12). Our findings may be explained by the timing of concussions relative to testing. In our study, athletes with recent concussions (within the last 3 months) were excluded, implying that participants included in our study had likely recovered from prior concussions they reported. Despite a substantial portion of athletes reporting a history of concussion, the cognitive demands assessed by NeuroTracker may not be sensitive to the lingering effects of prior concussions.

Our study yielded mixed results regarding the type of sport played. Examining NeuroTracker performance in isolation, male ice hockey players outperformed participants in all other sports. However, the overall average performance did not show significance. It is plausible that certain sports were underrepresented in terms of participants, suggesting that future studies should encompass larger athlete samples.

Acknowledging the potential underrepresentation of certain sports and the need for larger samples is critical. Future research should aim to include a more diverse range of sports, ensuring a comprehensive understanding of how various athletic activities may influence NeuroTracker performance. In conclusion our study demonstrated that biological sex and type of sport influence NeuroTracker performance in a sample of university level varsity athletes. These factors should be considered in future studies, especially when it comes to using the NeuroTracker as a tool to make informed decisions on certain populations, such as in the case of talent identification, for example.

## Data Availability

The data that support the findings of this study are available from the corresponding author (L-AC-B), upon reasonable request.

## References

[1] Faubert J Sidebottom L. Perceptual-cognitive training of athletes. J Clin Sport Psychol. (2012) 6(1):85–102. 10.1123/jcsp.6.1.85

[2] Corbin-Berrigan LA Kowalski K Faubert J Christie B Gagnon I. Three-dimensional multiple object tracking in the pediatric population: the NeuroTracker and its promising role in the management of mild traumatic brain injury. NeuroReport. (2018) 29(7):559–63. 10.1097/WNR.0000000000000988 29481522

[3] Deschamps A Giguère-Lemieux É Fait P Corbin-Berrigan LA. Test–retest reliability of the NeuroTracker compared to the impact test for the management of mild traumatic brain injuries during two consecutive university sport seasons. Brain Inj. (2022) 36(8):977–84. 10.1080/02699052.2022.2109738 35950219

[4] Mangine GT Wells AJ Hoffman JR. Visual tracking speed is related to basketball-specific measures of performance in NBA players. J Strength Cond Res. (2014) 9:2406–14. 10.1519/JSC.0000000000000550 24875429

[5] Tétreault É Fortin-Guichard D McArthur J Vigneault A Grondin S. About the predictive value of a 3D multiple object tracking device for talent identification in elite ice hockey players. Res Q Exerc Sport. (2023) 95(2):1–14. 10.1080/02701367.2023.2216266 37463224

[6] Vater C Gray R Holcombe AO. A critical systematic review of the NeuroTracker perceptual-cognitive training tool. Psychon Bull Rev. (2021):1–26. 10.3758/s13423-021-01892-2 PMC8500884 33821464

[7] Legault I Sutterlin-Guindon D Faubert J. Perceptual cognitive abilities in young athletes: a gender comparison. PLoS One. (2022) 17(8):e0273607. 10.1371/journal.pone.0273607 36044462 PMC9432702

[8] Alsalaheen B Stockdale K Pechumer D Giessing A He X Broglio SP. Cumulative effects of concussion history on baseline computerized neurocognitive test scores: systematic review and meta-analysis. Sports Health. (2017) 9(4):324–32. 10.1177/1941738117713974 28661827 PMC5496709

[9] Scheuringer A Pletzer B. Sex differences in the Kimchi-Palmer task revisited: global reaction times, but not number of global choices differ between adult men and women. Physiol Behav. (2016) 165:159–65. 10.1016/j.physbeh.2016.07.012 27445034 PMC7115960

[10] Roudaia E Faubert J. Different effects of aging and gender on the temporal resolution in attentional tracking. J Vis. (2017) 17(11):1. 10.1167/17.11.1 28862709

[11] Corbin-Berrigan L-A Faubert J Kowalski K. Three-dimensional multiple object tracking in the pediatric population: the NeuroTracker and its promising role in the management of mild traumatic brain injury. NeuroReport. (2018) 7:559–63. 10.1097/WNR.0000000000000988 29481522

[12] Corbin-Berrigan LA Kowalski K Faubert J Christie B Gagnon I. Could NeuroTracker be used as a clinical marker of recovery following pediatric mild traumatic brain injury? An exploratory study. Brain Inj. (2020) 34(3):385–9. 10.1080/02699052.2020.1723699 32013583

